# Laparoscopic Cholecystectomy for a Giant Gallstone: A Case Report

**DOI:** 10.7759/cureus.31546

**Published:** 2022-11-15

**Authors:** Mohammed Alfehaid

**Affiliations:** 1 Department of Surgery, Unaizah College of Medicine and Medical Sciences, Qassim University, Qassim, SAU

**Keywords:** umbilical port site, classical cholecystectomy, giant gallstone, cholelithiasis, laparoscopic cholecystectomy (lc)

## Abstract

Cholelithiasis is very common, affecting around 10-15% of the general population, but giant gallstones measuring 5 cm or more are very rare with only a few cases reported in the literature. Laparoscopic cholecystectomy (LC) is very difficult in such cases, especially in emergency situations, but can be safely performed by skilled surgeons. One such 47-year-old male with a giant gallstone measuring 8.7 cm x 4.2 cm x 3.4 cm was safely managed by laparoscopic approach and is presented due to its rarity.

## Introduction

Cholelithiasis (gallstones) is the commonest biliary pathology in most Westernized societies. In Saudi Arabia, the prevalence has been found to be 8.6-11.7% [1-2]. The majority of gallstones are asymptomatic (>80%) and approximately 1-2% per year tend to turn symptomatic with a low rate of complications. Gallstones larger than 5 cm in diameter are referred to as "giant gallstones." Only a small number of giant gallstones have been described in the literature, and they are rarely encountered [3]. Laparoscopic cholecystectomy (LC) has become the gold standard for the treatment of symptomatic gallstones but it is very challenging to remove giant gallstones by LC, especially in the emergency situations like acute cholecystitis. In this case report, one such case of giant gallstone is presented which was successfully managed laparoscopically in emergency settings.

## Case presentation

A 47-year-old male presented to the emergency department with a history of about 12 hours of pain in the right upper quadrant of the abdomen. The pain score was 7/10 on numerical rating scales (NRS) and radiated to the right scapular region. There was nausea and anorexia. In the past two to three years, the patient had experienced episodes of self-resolving post-prandial upper abdominal pain. He was otherwise not known to have any significant past history. On examination, blood pressure was 114/76 mmHg, pulse was 90 beats per minute, and temperature was 37.8 C. The right hypochondrium was tender and Murphy's sign of cholecystitis was positive. Laboratory tests showed an increased white blood cell count (WBC) of 9.4x10^9/L; other blood tests including urine analysis, liver function tests, and serum amylase were within normal limits.

**Figure 1:**
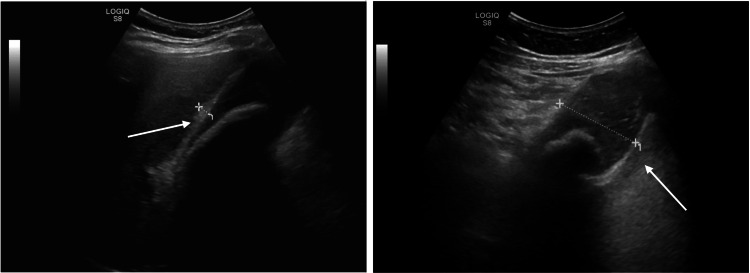
US Abdomen revealing a giant gallstone. Left arrow points toward the thickened gallbladder wall while the right arrow points to the giant gallstone.

Abdominal ultrasound revealed a distended gallbladder containing sludge and a single significant large calculus with wall thickening reaching 7mm and pericholecystic fluid (Figure 1).

The diagnosis of acute cholecystitis secondary to cholelithiasis was made and the patient was admitted to the hospital while the laparoscopic cholecystectomy was undertaken on the second day of admission.

**Figure 2:**
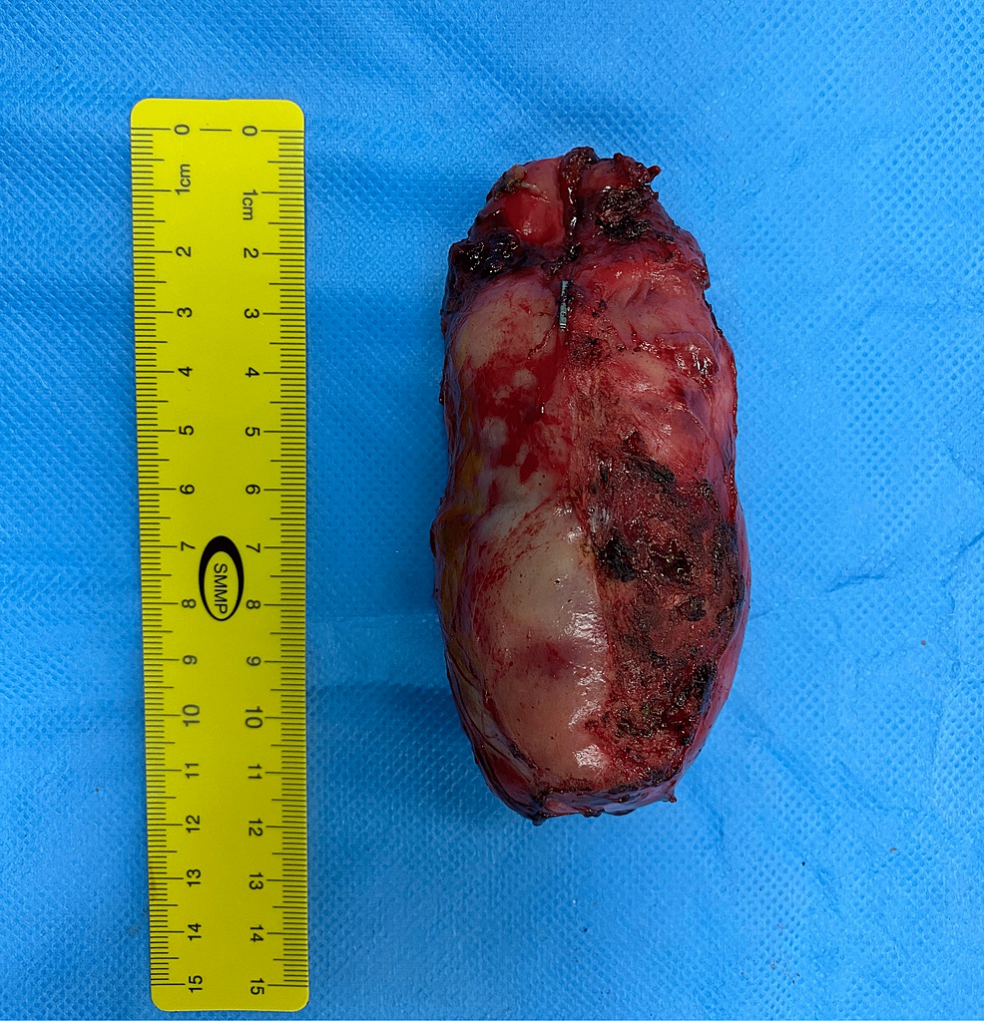
Excised gallbladder specimen

**Figure 3:**
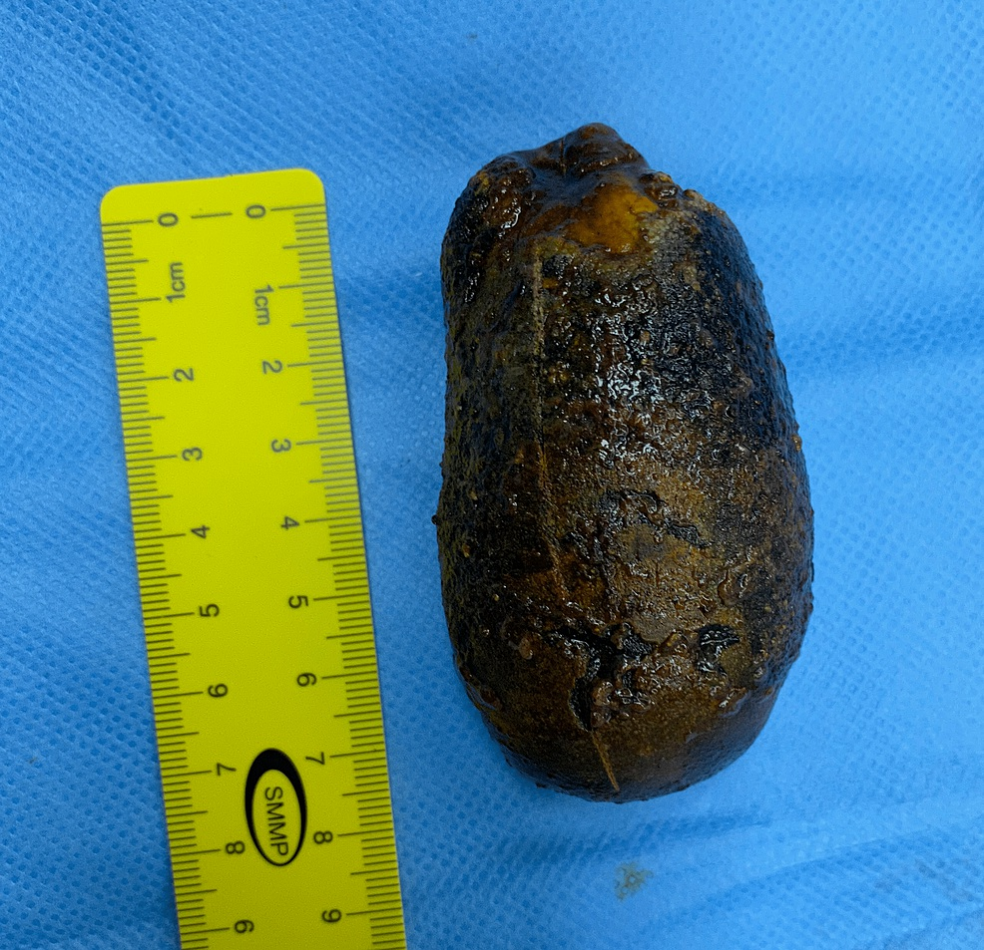
Giant gallstone measuring 8.7 cm in length.

**Figure 4:**
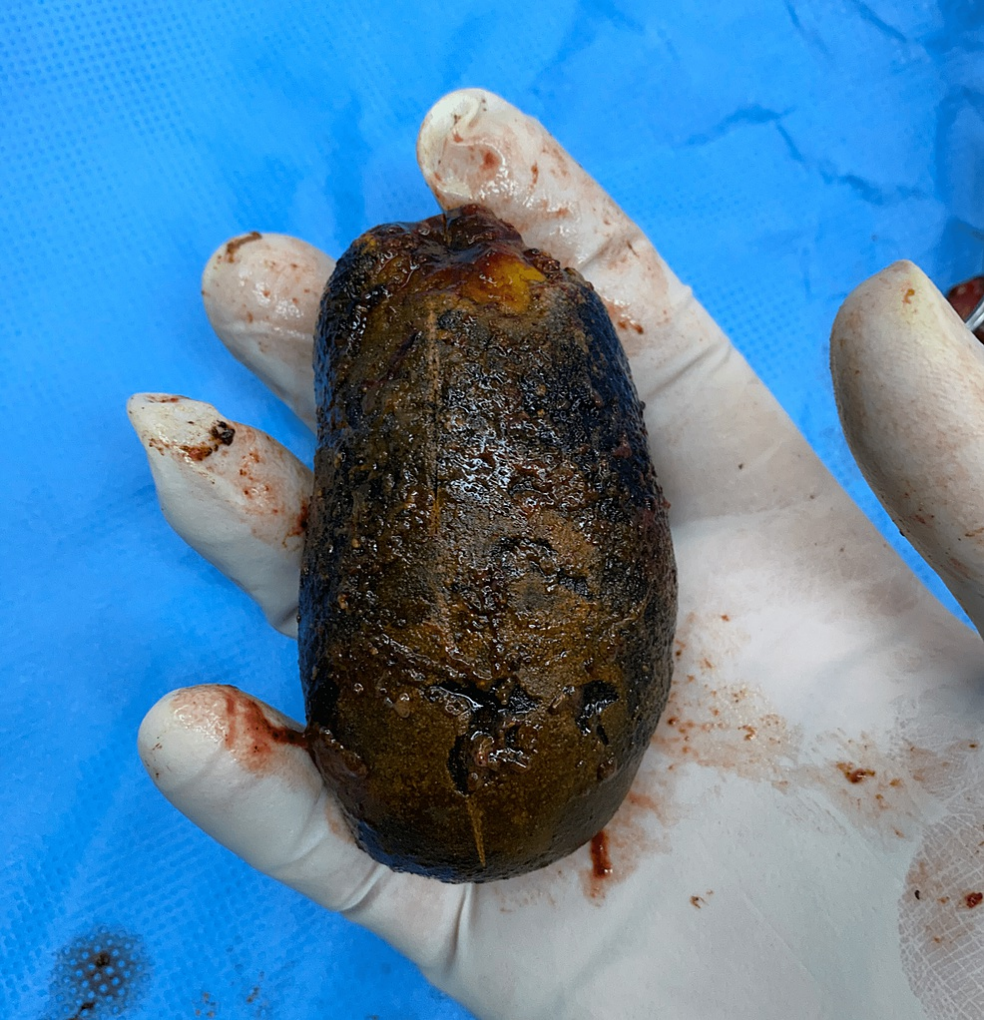
Giant gallstone held in surgeon's hand

At the operation, the patient was placed in the supine position and an 11 mm infra umbilical camera port was inserted after the creation of pneumoperitoneum with a Veress needle. One 10 mm epigastric working port with two additional 5 mm supporting ports were inserted under vision. There were minimal dense adhesions between the greater omentum and gallbladder and adhesiolysis was achieved with the help of electro-cautery. The gallbladder was nearly completely filled with the gallstone, making the wall tense and difficult to grasp using non-traumatic forceps. The critical view of safety was achieved and the clipping of the cystic artery and duct was done. The gallbladder was dissected off the cystic plate, put in an endo bag, and extracted out after the necessary extension of the infra umbilical incision. The gallbladder specimen measured 12 cm x 6.6 cm x 5.3 cm (Figure 2) and on the cut section, a solitary gallstone measuring 8.7 cm x 4.2 cm x 3.4 cm (178 grams) was recovered (Figure 3, 4).

The postoperative course was uneventful and the patient was discharged on the second postoperative day. The histopathological report showed features of acute on chronic follicular cholecystitis and no evidence of malignancy.

## Discussion

Cholelithiasis is one of the commonest surgical problems and is attributed to various factors including cholesterol hypersecretion, reduced excretion of bile salts and/or phospholipids bile salts, hypomotility of gallbladder, and haemolytic disorders. While 20-25 million people have gallstones in the USA, the majority of patients experience no symptoms and hence only 300,000 cholecystectomies are performed annually [4]. According to a study by Abu-Eshy et al., the frequency of gallstones diseases in Saudi Arabia was 8.6% in a demographic cohort living at high altitudes [1], whereas Alishi et al. found that the prevalence was 11.7% in Riyadh, the country's capital and largest metropolis [2]. Gallstones larger than 5 cm are referred to as "giant gallstones" and are rare [3].

Due to the proven relationship between the greater size of gallstones with gallbladder cancer, patients with giant gallstones even when asymptomatic are recommended cholecystectomy. In addition to that, it is considered a risk factor for fistula formation with the possibility of bowel obstruction and Mirrizzi syndrome [5]. And regarding the choice of surgical approach, many workers believe that a giant gallstone may be an indication for the adoption of an open approach [6] or else for conversion to open cholecystectomy from laparoscopic [7]. This is due to the difficulties that the giant stones pose during LC. First, the bigger stones usually cause inflammation which thickens the gallbladder wall. Bivariate analysis by Raman et al. has demonstrated a correlation between conversion to open surgery and gallbladder wall thickness [8]. Second, a large gallstone may cause technical challenges. For instance, it may be highly challenging to hold the gallbladder with laparoscopic tools and to obtain the proper anatomical exposure of Calot's triangle for safe dissection [9]. And towards the end of LC, at the time of the retrieval of the gallbladder specimen along with the giant stone, there is a size mismatch with the 10-11 mm port sites which require an extension of the incision. Hajibandeh et al. in a recent systematic review have shown that gallbladder specimen retrieval through the umbilical port retrieval takes lesser time and may be associated with less postoperative pain in patients in comparison to retrieval through the epigastric port retrieval [10]. In the present case report also, the author resorted to an extension of umbilical port site incision.

However, even with the giant gallstones, LC performed by a skilled laparoscopic surgeon is the best initial course of action, in the opinion of the author, unless the technical challenges or an inability to expose the anatomy led to conversion to open cholecystectomy. The author was able to conduct safe LC without any conversion even in emergency settings. Similar experiences have been reported in other published case reports in the peer-reviewed literature [3,9,11-12].

Different-sized giant gallstones have been reported in the literature and most of them are solitary. Singh et al. [12] removed a giant gallstone measuring 12.8 cm x 7 cm and this is mentioned as the largest gallstone removed laparoscopically in the world. Becerra et al. [13] reported the removal of a 16.8 cm x 7.8 cm x 4.1 cm (278.0 g) gallstone by the classical open cholecystectomy. Igwe and Diri [4] in their report of two cases operated laparoscopically, had a solitary gallstone gallbladder calculus measuring 8.2cm x 7.5 cm in the first case whereas in the second case, there were multiple calculi with one measuring 8 cm x 6 cm.

## Conclusions

Giant gallstones are rare and although they pose technical challenges, laparoscopic cholecystectomy can be safely carried out by skilled surgeons and specimen retrieval may be achieved by extension of umbilical port site incision. However, if the anatomy cannot be properly delineated, conversion to open cholecystectomy is a safe management strategy.
